# Future perspectives of surgical treatment of breast cancer

**DOI:** 10.1016/j.amsu.2020.09.021

**Published:** 2020-09-16

**Authors:** Margit Riis

**Affiliations:** a Department of Oncology, Section of Breast- and Endocrine Surgery, Oslo University Hospital, Oslo, Norway; b Department of Cancer Genetics, Institute for Cancer Research, The Norwegian Radium Hospital, Oslo University Hospital, Oslo, Norway

## Abstract

Breast cancer is a heterogenous disease which requires updates on scientific research to offer patients the best possible and personalized treatment. Modern surgical treatment of breast cancer is recently been reviewed and it is clearly a field of continuous change and improvement. The change in treatment strategies is a challenge given that prognosis is already acceptable. The collaboration between specialties across different countries is needed to exchange experience and improve international guidelines. Multidisciplinary teams are mandatory for the patient to be provided with all treatment options and to make an informed decision on their treatment. The major trend in surgical treatment of breast cancer is de-escalating surgery and more focus on tumor biology. A “one-size-fits all” approach does not apply in treatment of breast cancer today. There are two major questions in future aspects of breast cancer treatment; Can surgery in the breast be omitted in patients with a pathological complete response after neoadjuvant therapy? And can some patients be spared axillary surgery? The timing of surgery is also debatable. These are major questions that need to be answered and through randomized controlled trials. This commentary gives an insight on thoughts and scientific research with focus on future aspects of breast cancer treatment.

Breast cancer surgery has developed rapidly the last four decades [1]. From being major surgical challenges, like Halsted's mastectomy which was removal of the breast, en bloc with the pectoralis muscle and the axillary lymph nodes in addition to a great deal of skin to minimal resections in breast conserving therapy [2]. In the axilla the extent of surgery has been reduced dramatically, again from the Halstet's procedure where lymph nodes were resected from all levels, level I, II, and III, to procedures of today which may be no surgery at all in the axilla. There was a publication in 2001 with the focus on surgical aspects on breast cancer treatment where current standards and future perspectives were discussed [3]. Other publications have focused on milestones of breast cancer treatment both medically and surgically [1,[4], [5], [6]]. Already from the initiation of breast conserving therapy de-escalation of surgery was a major subject, both in the axilla and in the breast. Multidisciplinary teams were introduced as an absolute necessary part of breast cancer treatment. Twenty years have passed and the same issues are still in focus [1].

Molecular subtypes were introduced in the millennium change [7,8], with special focus on the impact on prognosis and introduction into the clinic [9]. De-escalation of the medical treatment of breast cancer is an issue, but in this case, we focus on personalized treatment. The integration of gene expression and molecular profiling of breast tumors have revealed the molecular subtypes which are the basis of personalized treatment [7,10,11]. Two prospective randomized controlled trials have changed the medical treatment from being based on clinical parameters to include genomic parameters in addition to clinical parameters in the decision making for optimal medical treatment. The phase III EORTC 10041/BIG 3–04 Microarray in Node-Negative and 1 to 3 Positive Lymph Node Disease May Avoid Chemotherapy (MINDACT) trial (NCT00433589) was an international, prospective, randomized study evaluating the clinical utility of the 70-gene expression signature (MammaPrint®) combined with clinical-pathological criteria for selection of patients for adjuvant chemotherapy in breast cancer [12,13]. TailorX was the corresponding prospective trial using OncotypeDX, a separate panel of involved genes [[14], [15], [16], [17], [18]]. The cost-effectivity of the 70 gene signatures has been evaluated in the beginning of the prospective studies [19], but also 10 years later [20]. In the latter paper, they used a hybrid decision tree-Markov model which simulated treatment strategies in accordance with the 70-gene signature with clinical assessment versus clinical assessment alone, over a 10-year time horizon [20]. They focused on estrogen receptor positive (ER+), human epidermal growth factor 2 negative (HER2-) patients, and six countries were involved. Treatments strategies guided by the 70 gene signatures had a greater score in quality-adjusted life years (QALYs), and cost of the 70 gene signature strategy were lower in five of the six countries. They concluded that using the 70 gene signature was safe in guiding chemotherapy in clinical high risk patients in this selected group of patients, and it was cost-effective compared to using solely clinical parameters ().

Neoadjuvant chemotherapy (NACT) was initially introduced to downstage locally advanced breast cancers and to allow surgery in primarily inoperable cases. This has changed into also including patients with initially operable breast cancer, but where the neoadjuvant treatment is preferred to downstage the disease for the possibility of reducing tumor size and in that perspective allow for lesser extent of surgery in the breast. As we have shown in the present review [1] sentinel node biopsy is safe and feasible both in patients going for primary surgery and those having neoadjuvant therapy. The timing of sentinel node biopsy differs among the different institutions and regions. The argument for doing the sentinel node biopsy prior to neoadjuvant therapy is that the untreated lymph node status is still considered as one of the strongest prognostic factors. It is important to keep in mind that adjuvant radiotherapy is based on the primary lymph node status, while the extent of systemic treatment is decided rather by tumor biology and predictive factors [21].

There are basically three arguments in favor of doing the sentinel node biopsy after neoadjuvant therapy. (1) The most obvious reason is that the patient is spared an extra surgical procedure. (2) Pathological complete response (pCR) is a parameter to study the effect of neoadjuvant therapy. This is a strong predictor of overall survival and is assessed both in the breast and the axilla. (3) The last argument for sentinel node biopsy after neoadjuvant therapy is the knowledge of 20–40% of the patients will convert from cN0pN1 stage to ypN0 (clinical node negative but pathological node positive to pathological node negative), and these patients might be spared regional treatment to the axilla [21].

Tumor biology and molecular subtype of the breast cancer is increasingly being implicated in the medical treatment of breast cancer, but can it also be implied in the surgical treatment? Triple negative and Her2 positive breast cancer generally respond better to neoadjuvant chemotherapy than those that are hormone receptor positive, that is basal-like and Her2 enriched respond better than luminal breast cancer [[22], [23], [24]]. Luminal breast cancers however can be reduced in size by neoadjuvant endocrine therapy, both traditional endocrine therapy like tamoxifen and aromatase inhibitors, but also modern treatment like CDK4/6 inhibitors [25]. Future studies are needed to evaluate if axillary node dissection can be replaced by radiotherapy in patients with clinical node negative but pathological node positive disease. This is the case for around 25% of clinically node negative patients. The results from the Z0011 and AMAROS trial led to the omission of complete axillary dissection in primary operable breast cancer patients, showing that axillary radiation was as good in terms of locoregional recurrence but with significantly less morbidity [26,27]. The accuracy of sentinel node biopsy after neoadjuvant chemotherapy was addressed by a review from a Dutch scientist group [28]. Results were not uniform across all including studies, and not enough evidence to recommend this as a standard surgical procedure. They stress however the importance of biology and to guide treatment based on the heterogeneity of the breast cancer.

There are two major questions in the future perspective of surgical treatment of breast cancer.

(1) Can surgery in the breast be omitted in patients who have a pCR
(2) Can some patients be spared all axillary surgery, both for staging and treatment?

These are major questions but due to ethical consideration difficult to be studied in prospective randomized controlled studies. This stresses the importance of international multicenter prospective studies which may aid in consensus of common guidelines for institutions treating breast cancer. More importantly it is important to implement the though of “one size fits all” does not apply for breast cancer patients.

## Provenance and peer review

Not commissioned, externally peer reviewed.
